# (2*Z*,3*E*)-2-{[1-(4-Chloro­benz­yl)-1*H*-indol-3-yl]methyl­idene}quinuclidin-3-one oxime

**DOI:** 10.1107/S160053681100612X

**Published:** 2011-02-26

**Authors:** Narsimha Reddy Penthala, Thirupathi Reddy Yerram Reddy, Sean Parkin, Peter A. Crooks

**Affiliations:** a Department of Pharmaceutical Sciences, College of Pharmacy, University of Kentucky, Lexington, KY 40536, USA; b Department of Chemistry, University of Kentucky, Lexington, KY 40506, USA

## Abstract

In the title compound, C_23_H_22_ClN_3_O, the benzene ring of the 4-chorobenzyl group makes a dihedral angle of 78.56 (6)° with the best plane of the indole ring. The double bond connecting the aza­bicyclic and indole groups adopts a *Z* geometry. The geometry adopted by the C=N bond with respect to the N—OH bond is *trans*. The absolute configuration of the compound was determined from refinement of the Flack parameter.

## Related literature

For 2-indol-3-yl-methyl­enequinuclidin-3-ols and NADPH oxidase activity, see: Sekhar *et al.* (2003 ▶) and for novel substituted (*Z*)-2-(*N*-benzyl­indol-3-yl­methyl­ene)quinuclidin-3-one and (*Z*)-(±)-2-(*N*-benzyl­indol-3-yl­methyl­ene)quinuc­lidin-3-ol derivatives as potent thermal sensitizing agents, see: Sonar *et al.* (2007 ▶). For di- and triindolyl­methanes: mol­ecular structures, see: Mason *et al.* (2003 ▶) and for structures of 1*H*-indole-3-ethyl­ene-3′-meth­oxy­salicylaldimine and 3-[3′-aza­pentyl-3′-en-4′-(2′′-hy­droxy­phen­yl)]indole, see: Zarza *et al.* (1988 ▶). For the radio-sensitization activity associated with *N*-benzyl­indolyl-1-aza­bicyclo­[2.2.2]octan-3-ones, see: Sonar *et al.* (2003 ▶).
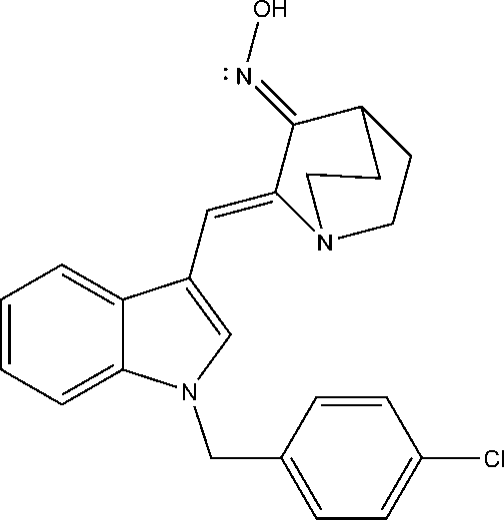


## Experimental

### 

#### Crystal data

C_23_H_22_ClN_3_O
*M* *_r_* = 391.89
Orthorhombic, 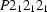

*a* = 5.8382 (1) Å
*b* = 10.7005 (2) Å
*c* = 30.9451 (6) Å
*V* = 1933.19 (6) Å^3^
*Z* = 4
Mo *K*α radiation
μ = 0.22 mm^−1^
*T* = 90 K
0.40 × 0.12 × 0.08 mm

#### Data collection

Nonius KappaCCD diffractometer
Absorption correction: multi-scan (*SCALEPACK*; Otwinowski & Minor, 1997 ▶) *T*_min_ = 0.918, *T*_max_ = 0.983
37270 measured reflections
4433 independent reflections
3150 reflections with *I* > 2σ(*I*)
*R*_int_ = 0.103

#### Refinement

*R*[*F* ^2^ > 2σ(*F* ^2^)] = 0.049
*wR*(*F* ^2^) = 0.137
*S* = 1.06
4433 reflections
254 parameters
H-atom parameters constrained
Δρ_max_ = 0.40 e Å^−3^
Δρ_min_ = −0.24 e Å^−3^
Absolute structure: Flack (1983 ▶), 1853 Friedel pairs
Flack parameter: −0.03 (4)

### 

Data collection: *COLLECT* (Nonius, 1998 ▶); cell refinement: *SCALEPACK* (Otwinowski & Minor, 1997 ▶); data reduction: *DENZO-SMN* (Otwinowski & Minor, 1997 ▶); program(s) used to solve structure: *SHELXS97* (Sheldrick, 2008 ▶); program(s) used to refine structure: *SHELXL97* (Sheldrick, 2008 ▶); molecular graphics: *XP* in *SHELXTL* (Sheldrick, 2008 ▶); software used to prepare material for publication: *SHELXL97* and local procedures.

## Supplementary Material

Crystal structure: contains datablocks global, I. DOI: 10.1107/S160053681100612X/fj2375sup1.cif

Structure factors: contains datablocks I. DOI: 10.1107/S160053681100612X/fj2375Isup2.hkl

Additional supplementary materials:  crystallographic information; 3D view; checkCIF report

## Figures and Tables

**Fig. 1.**
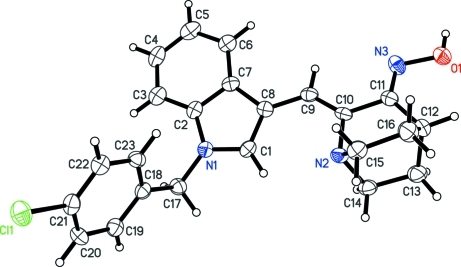
A view of the molecule with the atom numbering scheme. Displacement ellipsoids are drawn at the 50% probability level.

## supplementary crystallographic information

### Comment

In view of the radio-sensitization activity associated with *N*-benzylindolyl-1-azabicyclo[2.2.2]octan-3-ones (Sonar *et al.*, 2007), we have undertaken the synthesis and structural analysis of a series of (2*Z*,3*E*)-2-((1-benzyl-1*H*-indol-3-yl)methylene) quinuclidin-3-one oximes. Systematic structural modification of the active molecule (*Z*)-2-(1-benzyl-1*H*-indol-3-ylmethylene)1- azabicyclo[2.2.2]octan-3-ol, was carried out, and the title compound was synthesized as its structural analogue. The X-ray analysis of the title compound was carried out to confirm the double-bond geometry of the molecule, and to determine the molecular conformation in the crystal structure.

X-ray crystallography confirmed the molecular structure and atom connectivity for title compound, as illustrated in Fig. 1. The indole ring is planar, with bond distances and angles comparable with those previously reported for other indole derivatives (Mason *et al.*., 2003; Zarza *et al.*., 1988). The benzene ring of the benzyl group linked to the N1 position of the indole ring is slightly twisted, making a dihedral angle of 78.56 (6)° with the plane of the indole ring system.

The title compound is the *Z* isomer, with the C10—C11 bond in a *trans* disposition with respect to the C8—C9 bond. The double bond has a nearly planar arrangement, since the r.m.s. deviation from the best plane passing through atoms N2/C10/ C11/C9/C8 is 0.0143 (15) Å. The azabicyclic system presents very small distortions around atoms N2, C14, C13, C12, C16 and C11. The value of the C1=C8—C9=C10 torsion angle -13.87° indicates the deviation of the indole ring from the plane of the double bond connected to the azabicyclic ring.

### Experimental

Compound 2-((1-(4-chlorobenzyl)-1*H*-indol-3-yl)methylene) quinuclidin-3-one was prepared by aldol condensation of 1-(4-chlorobenzyl-indole-3-carboxaldehyde with 1-azabicyclo[2.2.2]octan- 3-one to afford (*Z*)-2-(1-(4-chlorobenzyl-1*H*-indol-3-yl methylene)-1-azabicyclo[2.2.2]octan-3-one, as a single geometric isomer, according to the previously reported procedure of Sonar *et al.* (2003). A mixture (*Z*)-2-(1-(4-chlorobenzyl-1*H*-indol-3-yl methylene)1-azabicyclo[2.2.2]octan-3-one (0.5 g, 1.32 mmol), hydroxylamine hydrocloride (0.18 g, 2.65 mmol) and sodium acetate trihydrate (0.36 g, 2.65 mmol) was stirred in methanol (25 ml) under reflux for 8 hrs. The reaction mixture was cooled to room temperature, diluted with water (15 ml), and the light yellow solid that separated was collected by filtration, washed with water and dried, to afford the the crude product. Crystallization from methanol gave a colorless crystalline product of (2*Z*,3*E*)-2- ((1-(4-chlorobenzyl)-1*H*-indol-3-yl)methylene) quinuclidin-3-one oxime that was suitable for X-ray analysis. ^1^H NMR (CDCl_3_): δ 1.76–1.79 (*m*, 4H), 2.91–3.09 (*m*, 4H), 3.67–3.69 (*m*, 1H), 5.34 (*s*, 2H), 7.01–7.04 (*d*, 2H), 7.10 (*s*, 1H), 7.14–7.25 (*m*, 5H), 7.30 (*bs*, 1H), 7.79–7.83 (*d*, 1H), 8.21 (*s*, 1H) *p.p.m.*; ^13^C NMR (DMSO d_6_): δ 24.46, 25.87, 47.77, 50.06, 109.92, 110.45, 111.47, 119.34, 120.34, 122.39, 128.05, 128.63, 129.09, 131.27, 133.54, 135.80, 135.98, 138.23, 161.31 *p.p.m.*.

### Refinement

H atoms were found in difference Fourier maps and subsequently placed in idealized positions with constrained distances of 0.99 Å (*R*_2_CH_2_), 1.00 Å (*R*_3_CH), 0.95 Å (C_Ar_H), 0.84 Å (O—H), and with *U*_iso_(H) values set to either 1.2*U*_eq_ or 1.5*U*_eq_ (OH) of the attached atom.

### Crystal data

**Table:**

C_23_H_22_ClN_3_O	*F*(000) = 824
*M**_r_* = 391.89	*D*_x_ = 1.346 Mg m^−^^3^
Orthorhombic, *P*2_1_2_1_2_1_	Mo *K*α radiation, λ = 0.71073 Å
Hall symbol: P 2ac 2ab	Cell parameters from 2610 reflections
*a* = 5.8382 (1) Å	θ = 1.0–27.5°
*b* = 10.7005 (2) Å	µ = 0.22 mm^−^^1^
*c* = 30.9451 (6) Å	*T* = 90 K
*V* = 1933.19 (6) Å^3^	Plate, colourless
*Z* = 4	0.40 × 0.12 × 0.08 mm

### Data collection

**Table:**

Nonius KappaCCD diffractometer	4433 independent reflections
Radiation source: fine-focus sealed tube	3150 reflections with *I* > 2σ(*I*)
graphite	*R*_int_ = 0.103
Detector resolution: 9.1 pixels mm^-1^	θ_max_ = 27.5°, θ_min_ = 1.3°
ω scans at fixed χ = 55°	*h* = −7→7
Absorption correction: multi-scan (*SCALEPACK*; Otwinowski & Minor, 1997)	*k* = −13→13
*T*_min_ = 0.918, *T*_max_ = 0.983	*l* = −39→40
37270 measured reflections	

### Refinement

**Table:**

Refinement on *F*^2^	Secondary atom site location: difference Fourier map
Least-squares matrix: full	Hydrogen site location: inferred from neighbouring sites
*R*[*F*^2^ > 2σ(*F*^2^)] = 0.049	H-atom parameters constrained
*wR*(*F*^2^) = 0.137	*w* = 1/[σ^2^(*F*_o_^2^) + (0.0788*P*)^2^] where *P* = (*F*_o_^2^ + 2*F*_c_^2^)/3
*S* = 1.06	(Δ/σ)_max_ < 0.001
4433 reflections	Δρ_max_ = 0.40 e Å^−^^3^
254 parameters	Δρ_min_ = −0.24 e Å^−^^3^
0 restraints	Absolute structure: Flack (1983), 1853 Friedel pairs
Primary atom site location: structure-invariant direct methods	Flack parameter: −0.03 (4)

### Special details

**Table:**

Geometry. All e.s.d.'s (except the e.s.d. in the dihedral angle between two l.s. planes) are estimated using the full covariance matrix. The cell e.s.d.'s are taken into account individually in the estimation of e.s.d.'s in distances, angles and torsion angles; correlations between e.s.d.'s in cell parameters are only used when they are defined by crystal symmetry. An approximate (isotropic) treatment of cell e.s.d.'s is used for estimating e.s.d.'s involving l.s. planes.
Refinement. Refinement of *F*^2^ against ALL reflections. The weighted *R*-factor *wR* and goodness of fit *S* are based on *F*^2^, conventional *R*-factors *R* are based on *F*, with *F* set to zero for negative *F*^2^. The threshold expression of *F*^2^ > 2σ(*F*^2^) is used only for calculating *R*-factors(gt) *etc*. and is not relevant to the choice of reflections for refinement. *R*-factors based on *F*^2^ are statistically about twice as large as those based on *F*, and *R*- factors based on ALL data will be even larger.

### Fractional atomic coordinates and isotropic or equivalent isotropic displacement parameters (Å^2^)

**Table:**

	*x*	*y*	*z*	*U*_iso_*/*U*_eq_	
N1	0.1498 (4)	0.0866 (2)	0.89083 (7)	0.0245 (5)	
N2	0.0660 (4)	0.1260 (2)	0.75130 (7)	0.0265 (5)	
N3	0.3789 (4)	0.4000 (2)	0.71465 (7)	0.0311 (6)	
O1	0.3831 (4)	0.45515 (18)	0.67293 (6)	0.0327 (5)	
H1O	0.4731	0.5164	0.6730	0.049*	
Cl1	0.28052 (15)	−0.51575 (6)	0.95786 (2)	0.0408 (2)	
C1	0.1254 (5)	0.1143 (3)	0.84757 (8)	0.0263 (6)	
H1	0.0091	0.0816	0.8293	0.032*	
C2	0.3379 (5)	0.1501 (2)	0.90706 (9)	0.0257 (6)	
C3	0.4304 (5)	0.1521 (2)	0.94836 (9)	0.0313 (7)	
H3	0.3656	0.1040	0.9711	0.038*	
C4	0.6191 (5)	0.2263 (3)	0.95518 (10)	0.0338 (7)	
H4	0.6823	0.2314	0.9834	0.041*	
C5	0.7198 (5)	0.2941 (2)	0.92199 (9)	0.0357 (7)	
H5	0.8529	0.3425	0.9276	0.043*	
C6	0.6283 (5)	0.2917 (2)	0.88076 (9)	0.0303 (7)	
H6	0.6975	0.3385	0.8582	0.036*	
C7	0.4340 (5)	0.2203 (2)	0.87271 (9)	0.0252 (6)	
C8	0.2938 (5)	0.1964 (2)	0.83457 (8)	0.0241 (6)	
C9	0.3253 (5)	0.2570 (2)	0.79299 (9)	0.0249 (6)	
H9	0.4310	0.3245	0.7925	0.030*	
C10	0.2250 (5)	0.2300 (2)	0.75544 (8)	0.0232 (6)	
C11	0.2554 (5)	0.2987 (2)	0.71447 (8)	0.0254 (6)	
C12	0.1228 (5)	0.2401 (3)	0.67821 (9)	0.0307 (7)	
H12	0.1483	0.2857	0.6504	0.037*	
C13	−0.1316 (5)	0.2419 (3)	0.69126 (10)	0.0371 (7)	
H13A	−0.2244	0.1972	0.6694	0.044*	
H13B	−0.1872	0.3292	0.6931	0.044*	
C14	−0.1551 (5)	0.1767 (3)	0.73590 (9)	0.0306 (7)	
H14A	−0.2140	0.2376	0.7573	0.037*	
H14B	−0.2677	0.1078	0.7336	0.037*	
C15	0.1538 (5)	0.0380 (2)	0.71842 (8)	0.0297 (7)	
H15A	0.0415	−0.0303	0.7143	0.036*	
H15B	0.2984	0.0004	0.7289	0.036*	
C16	0.1976 (6)	0.1027 (3)	0.67474 (9)	0.0355 (7)	
H16A	0.3624	0.0980	0.6674	0.043*	
H16B	0.1096	0.0603	0.6517	0.043*	
C17	−0.0065 (5)	0.0109 (2)	0.91598 (9)	0.0283 (6)	
H17A	−0.1531	0.0046	0.9001	0.034*	
H17B	−0.0380	0.0546	0.9435	0.034*	
C18	0.0755 (5)	−0.1200 (3)	0.92624 (8)	0.0256 (6)	
C19	−0.0692 (5)	−0.1968 (3)	0.95003 (8)	0.0289 (6)	
H19	−0.2120	−0.1649	0.9597	0.035*	
C20	−0.0099 (5)	−0.3190 (2)	0.95994 (9)	0.0295 (6)	
H20	−0.1105	−0.3710	0.9760	0.035*	
C21	0.1993 (5)	−0.3631 (2)	0.94579 (9)	0.0291 (7)	
C22	0.3478 (5)	−0.2890 (2)	0.92239 (8)	0.0292 (7)	
H22	0.4916	−0.3209	0.9131	0.035*	
C23	0.2838 (5)	−0.1665 (2)	0.91249 (8)	0.0263 (6)	
H23	0.3841	−0.1148	0.8962	0.032*	

### Atomic displacement parameters (Å^2^)

**Table:**

	*U*^11^	*U*^22^	*U*^33^	*U*^12^	*U*^13^	*U*^23^
N1	0.0254 (13)	0.0248 (12)	0.0232 (12)	0.0017 (10)	0.0011 (10)	−0.0014 (9)
N2	0.0248 (13)	0.0252 (12)	0.0297 (13)	−0.0052 (11)	0.0008 (11)	0.0003 (10)
N3	0.0348 (15)	0.0266 (12)	0.0321 (13)	0.0075 (11)	0.0085 (12)	0.0067 (10)
O1	0.0341 (13)	0.0300 (11)	0.0340 (11)	−0.0025 (9)	0.0030 (9)	0.0037 (8)
Cl1	0.0491 (5)	0.0268 (4)	0.0464 (4)	0.0042 (4)	0.0006 (4)	0.0036 (3)
C1	0.0252 (15)	0.0261 (13)	0.0277 (15)	0.0036 (12)	−0.0044 (12)	−0.0025 (12)
C2	0.0287 (17)	0.0232 (14)	0.0252 (14)	0.0076 (12)	−0.0020 (12)	−0.0046 (11)
C3	0.0337 (17)	0.0270 (15)	0.0332 (17)	0.0081 (13)	−0.0022 (14)	−0.0056 (12)
C4	0.0417 (19)	0.0278 (14)	0.0318 (16)	0.0108 (14)	−0.0103 (15)	−0.0074 (13)
C5	0.0348 (18)	0.0264 (14)	0.0460 (18)	0.0022 (14)	−0.0096 (15)	−0.0120 (13)
C6	0.0320 (17)	0.0222 (14)	0.0367 (17)	0.0010 (13)	−0.0031 (14)	−0.0031 (12)
C7	0.0217 (15)	0.0228 (14)	0.0311 (15)	0.0027 (12)	0.0008 (12)	−0.0048 (12)
C8	0.0264 (15)	0.0173 (12)	0.0287 (14)	0.0014 (12)	0.0010 (12)	−0.0016 (10)
C9	0.0198 (14)	0.0208 (12)	0.0342 (15)	0.0015 (12)	0.0011 (12)	0.0005 (11)
C10	0.0175 (14)	0.0199 (12)	0.0323 (15)	−0.0013 (12)	0.0050 (12)	0.0021 (11)
C11	0.0221 (15)	0.0217 (13)	0.0325 (15)	0.0015 (12)	0.0029 (13)	0.0031 (11)
C12	0.0309 (17)	0.0337 (16)	0.0275 (16)	−0.0038 (14)	0.0004 (13)	0.0065 (12)
C13	0.0264 (17)	0.0386 (17)	0.0462 (18)	−0.0001 (14)	−0.0041 (15)	0.0116 (14)
C14	0.0216 (16)	0.0336 (15)	0.0368 (17)	−0.0003 (13)	−0.0009 (13)	0.0009 (13)
C15	0.0326 (17)	0.0222 (13)	0.0342 (16)	−0.0013 (13)	−0.0030 (13)	−0.0045 (12)
C16	0.0412 (19)	0.0346 (16)	0.0306 (16)	−0.0054 (15)	0.0029 (14)	−0.0057 (13)
C17	0.0265 (16)	0.0286 (15)	0.0298 (15)	0.0020 (13)	0.0035 (12)	0.0011 (12)
C18	0.0261 (15)	0.0285 (14)	0.0224 (14)	0.0010 (13)	−0.0023 (12)	−0.0018 (11)
C19	0.0289 (16)	0.0324 (15)	0.0254 (15)	0.0032 (13)	0.0012 (13)	−0.0002 (12)
C20	0.0311 (17)	0.0284 (14)	0.0288 (15)	−0.0034 (13)	0.0066 (13)	0.0033 (13)
C21	0.0377 (18)	0.0229 (13)	0.0268 (14)	0.0035 (13)	−0.0051 (13)	−0.0020 (11)
C22	0.0308 (17)	0.0297 (15)	0.0270 (15)	0.0041 (13)	−0.0003 (13)	−0.0038 (12)
C23	0.0255 (16)	0.0289 (14)	0.0247 (14)	−0.0023 (13)	0.0022 (13)	−0.0009 (11)

### Geometric parameters (Å, °)

**Table:**

N1—C1	1.379 (3)	C11—C12	1.500 (4)
N1—C2	1.385 (4)	C12—C16	1.538 (4)
N1—C17	1.448 (3)	C12—C13	1.539 (4)
N2—C10	1.455 (3)	C12—H12	1.0000
N2—C15	1.479 (3)	C13—C14	1.554 (4)
N2—C14	1.479 (4)	C13—H13A	0.9900
N3—C11	1.302 (4)	C13—H13B	0.9900
N3—O1	1.420 (3)	C14—H14A	0.9900
O1—H1O	0.8400	C14—H14B	0.9900
Cl1—C21	1.741 (3)	C15—C16	1.540 (4)
C1—C8	1.378 (4)	C15—H15A	0.9900
C1—H1	0.9500	C15—H15B	0.9900
C2—C3	1.387 (4)	C16—H16A	0.9900
C2—C7	1.418 (4)	C16—H16B	0.9900
C3—C4	1.374 (4)	C17—C18	1.514 (4)
C3—H3	0.9500	C17—H17A	0.9900
C4—C5	1.388 (4)	C17—H17B	0.9900
C4—H4	0.9500	C18—C23	1.381 (4)
C5—C6	1.383 (4)	C18—C19	1.389 (4)
C5—H5	0.9500	C19—C20	1.387 (4)
C6—C7	1.390 (4)	C19—H19	0.9500
C6—H6	0.9500	C20—C21	1.380 (4)
C7—C8	1.459 (4)	C20—H20	0.9500
C8—C9	1.453 (4)	C21—C22	1.381 (4)
C9—C10	1.333 (3)	C22—C23	1.397 (4)
C9—H9	0.9500	C22—H22	0.9500
C10—C11	1.476 (3)	C23—H23	0.9500
			
C1—N1—C2	109.2 (2)	C12—C13—H13A	110.1
C1—N1—C17	125.3 (2)	C14—C13—H13A	110.1
C2—N1—C17	125.4 (2)	C12—C13—H13B	110.1
C10—N2—C15	109.1 (2)	C14—C13—H13B	110.1
C10—N2—C14	107.7 (2)	H13A—C13—H13B	108.4
C15—N2—C14	108.3 (2)	N2—C14—C13	112.0 (2)
C11—N3—O1	110.6 (2)	N2—C14—H14A	109.2
N3—O1—H1O	109.5	C13—C14—H14A	109.2
C8—C1—N1	110.3 (2)	N2—C14—H14B	109.2
C8—C1—H1	124.9	C13—C14—H14B	109.2
N1—C1—H1	124.9	H14A—C14—H14B	107.9
N1—C2—C3	130.6 (3)	N2—C15—C16	112.0 (2)
N1—C2—C7	107.6 (2)	N2—C15—H15A	109.2
C3—C2—C7	121.9 (3)	C16—C15—H15A	109.2
C4—C3—C2	117.6 (3)	N2—C15—H15B	109.2
C4—C3—H3	121.2	C16—C15—H15B	109.2
C2—C3—H3	121.2	H15A—C15—H15B	107.9
C3—C4—C5	121.8 (3)	C12—C16—C15	108.8 (2)
C3—C4—H4	119.1	C12—C16—H16A	109.9
C5—C4—H4	119.1	C15—C16—H16A	109.9
C6—C5—C4	120.6 (3)	C12—C16—H16B	109.9
C6—C5—H5	119.7	C15—C16—H16B	109.9
C4—C5—H5	119.7	H16A—C16—H16B	108.3
C5—C6—C7	119.4 (3)	N1—C17—C18	115.6 (2)
C5—C6—H6	120.3	N1—C17—H17A	108.4
C7—C6—H6	120.3	C18—C17—H17A	108.4
C6—C7—C2	118.7 (3)	N1—C17—H17B	108.4
C6—C7—C8	134.4 (3)	C18—C17—H17B	108.4
C2—C7—C8	106.9 (2)	H17A—C17—H17B	107.4
C1—C8—C9	129.3 (3)	C23—C18—C19	119.0 (3)
C1—C8—C7	106.0 (2)	C23—C18—C17	123.2 (3)
C9—C8—C7	124.5 (2)	C19—C18—C17	117.8 (3)
C10—C9—C8	128.3 (3)	C20—C19—C18	121.5 (3)
C10—C9—H9	115.9	C20—C19—H19	119.2
C8—C9—H9	115.9	C18—C19—H19	119.2
C9—C10—N2	121.5 (2)	C21—C20—C19	118.2 (3)
C9—C10—C11	126.0 (2)	C21—C20—H20	120.9
N2—C10—C11	112.5 (2)	C19—C20—H20	120.9
N3—C11—C10	118.5 (2)	C20—C21—C22	121.7 (3)
N3—C11—C12	129.5 (2)	C20—C21—Cl1	119.6 (2)
C10—C11—C12	111.9 (2)	C22—C21—Cl1	118.7 (2)
C11—C12—C16	107.8 (2)	C21—C22—C23	119.0 (3)
C11—C12—C13	107.3 (2)	C21—C22—H22	120.5
C16—C12—C13	107.7 (3)	C23—C22—H22	120.5
C11—C12—H12	111.3	C18—C23—C22	120.4 (3)
C16—C12—H12	111.3	C18—C23—H23	119.8
C13—C12—H12	111.3	C22—C23—H23	119.8
C12—C13—C14	108.2 (2)		
			
C2—N1—C1—C8	−0.5 (3)	C9—C10—C11—N3	−4.8 (4)
C17—N1—C1—C8	175.3 (2)	N2—C10—C11—N3	174.5 (2)
C1—N1—C2—C3	−180.0 (3)	C9—C10—C11—C12	178.1 (3)
C17—N1—C2—C3	4.3 (4)	N2—C10—C11—C12	−2.7 (3)
C1—N1—C2—C7	0.2 (3)	N3—C11—C12—C16	127.3 (3)
C17—N1—C2—C7	−175.5 (2)	C10—C11—C12—C16	−55.9 (3)
N1—C2—C3—C4	−179.1 (3)	N3—C11—C12—C13	−117.0 (3)
C7—C2—C3—C4	0.7 (4)	C10—C11—C12—C13	59.8 (3)
C2—C3—C4—C5	−2.1 (4)	C11—C12—C13—C14	−54.6 (3)
C3—C4—C5—C6	1.9 (4)	C16—C12—C13—C14	61.1 (3)
C4—C5—C6—C7	−0.2 (4)	C10—N2—C14—C13	60.5 (3)
C5—C6—C7—C2	−1.1 (4)	C15—N2—C14—C13	−57.4 (3)
C5—C6—C7—C8	179.7 (3)	C12—C13—C14—N2	−3.5 (4)
N1—C2—C7—C6	−179.3 (2)	C10—N2—C15—C16	−55.6 (3)
C3—C2—C7—C6	0.9 (4)	C14—N2—C15—C16	61.4 (3)
N1—C2—C7—C8	0.1 (3)	C11—C12—C16—C15	57.8 (3)
C3—C2—C7—C8	−179.7 (2)	C13—C12—C16—C15	−57.6 (3)
N1—C1—C8—C9	−175.4 (2)	N2—C15—C16—C12	−3.0 (3)
N1—C1—C8—C7	0.5 (3)	C1—N1—C17—C18	104.6 (3)
C6—C7—C8—C1	178.9 (3)	C2—N1—C17—C18	−80.3 (3)
C2—C7—C8—C1	−0.4 (3)	N1—C17—C18—C23	−0.6 (4)
C6—C7—C8—C9	−5.0 (5)	N1—C17—C18—C19	−179.5 (2)
C2—C7—C8—C9	175.8 (2)	C23—C18—C19—C20	−0.5 (4)
C1—C8—C9—C10	−13.9 (5)	C17—C18—C19—C20	178.4 (2)
C7—C8—C9—C10	170.9 (3)	C18—C19—C20—C21	0.5 (4)
C8—C9—C10—N2	−2.2 (4)	C19—C20—C21—C22	0.0 (4)
C8—C9—C10—C11	176.9 (3)	C19—C20—C21—Cl1	179.6 (2)
C15—N2—C10—C9	−121.0 (3)	C20—C21—C22—C23	−0.5 (4)
C14—N2—C10—C9	121.6 (3)	Cl1—C21—C22—C23	179.9 (2)
C15—N2—C10—C11	59.7 (3)	C19—C18—C23—C22	0.0 (4)
C14—N2—C10—C11	−57.7 (3)	C17—C18—C23—C22	−178.9 (3)
O1—N3—C11—C10	−178.2 (2)	C21—C22—C23—C18	0.5 (4)
O1—N3—C11—C12	−1.6 (4)		
